# Development of Graphene‐Based Materials in Bone Tissue Engineaering

**DOI:** 10.1002/gch2.202100107

**Published:** 2021-12-02

**Authors:** Xiaoling Pan, Delin Cheng, Changshun Ruan, Yonglong Hong, Cheng Lin

**Affiliations:** ^1^ College of Stomatology Xinjiang Medical University Urumqi Xinjiang 830011 P. R. China; ^2^ Department of Oral Maxillofacial Surgery Shenzhen Hospital Southern Medical University Shenzhen 518000 P. R. China; ^3^ Research Center for Human Tissue and Organs Degeneration Institute of Biomedicine and Biotechnology Shenzhen Institutes of Advanced Technology Chinese Academy of Sciences Shenzhen 518055 P. R. China

**Keywords:** bone tissue engineering, CiteSpace, graphene, nanomedicine, scientometrics

## Abstract

Bone regeneration‐related graphene‐based materials (bGBMs) are increasingly attracting attention in tissue engineering due to their special physical and chemical properties. The purpose of this review is to quantitatively analyze mass academic literature in the field of bGBMs through scientometrics software CiteSpace, to demonstrate the rules and trends of bGBMs, thus to analyze and summarize the mechanisms behind the rules, and to provide clues for future research. First, the research status, hotspots, and frontiers of bGBMs are analyzed in an intuitively and vividly visualized way. Next, the extracted important subjects such as fabrication techniques, cytotoxicity, biodegradability, and osteoinductivity of bGBMs are presented, and the different mechanisms, in turn, are also discussed. Finally, photothermal therapy, which is considered an emerging area of application of bGBMs, is also presented. Based on this approach, this work finds that different studies report differing opinions on the biological properties of bGBMS due to the lack of consistency of GBMs preparation. Therefore, it is necessary to establish more standards in fabrication, characterization, and testing for bGBMs to further promote scientific progress and clinical translation.

## Graphical abstract

Herein, scientometrics is used to make a quantitative analysis of academic literature on bone regeneration‐related graphene‐based materials (bGBMs), to visually display the relevant research rules and trends. The fabrication techniques, cytotoxicity, biodegradability, osteoinductivity, photothermal properties, and thus the underlying mechanisms of bGBMs are discussed. Even with all these advantages, the lack of standardization of bGBM preparation technology remains a problem to be solved.

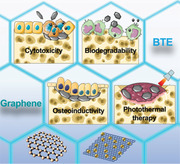

## 1 Introduction

Bone defect over a critical size, which often occurs as a result of traumatic injury, tumor resections, congenital defects, etc., could not be self‐healed by the body without additional aid.^[^ ^1^ ^]^ Currently, general treatments of critical‐sized bone defects include autografts, allografts, and the use of bone regeneration biomaterials.^[^ ^1^ ^]^ Autograft is the gold standard approach due to its satisfying osteogenicity. But it also faces drawbacks such as an invasive surgical operation for bone harvest and scarcity of available donor sites.^[^ ^2^ ^]^ Allograft is accompanied by the risk of disease transmission and immunological rejection.^[^ ^2^ ^]^ Therefore, significant efforts have been devoted to finding alternative approaches for bone regeneration. Bone tissue engineering (BTE) provides a promising alternative strategy to realize higher‐level restoration of bone defects. BTE exploits the elaborate combination of scaffolds, seeding cells, and biological factors to form a functional substitute or assist in situ regeneration. In most BTE research, stem cells are implanted into defective bone sites with the support of a biomaterial‐based scaffold and are supposed to proliferate and differentiate into osteoblasts to promote bone regeneration. However, the performances of many current BTE systems could not meet the therapeutic expectation due to problems such as insufficient osteogenic differentiation and limited vascularization.^[^ ^3^, ^4^, ^5^ ^]^ Many studies tried to improve the performance of BTE via enhancing the properties of the biomaterial‐based scaffold.^[^ ^6^ ^]^ In addition to traditional macro‐ or microscale forms of metals, ceramics, and polymers, their nanoscale counterparts have been extensively explored for BTE due to their unique properties.^[^ ^7^, ^8^, ^9^, ^10^ ^]^

Ever since Andre Geim and Konstantin Novoselov^[^ ^11^ ^]^ discovered a simple and feasible method for graphene isolation, graphene family materials (GFMs) have attracted great interest in regenerative medicine and tissue engineering. In BTE, bGBMs show promising potentials such as mechanical strength, high specific surface, and adsorption ability.^[^ ^12^, ^13^, ^14^, ^15^, ^16^, ^17^ ^]^ According to the composition, bGBMs could be categorized into GFMs and GFMs‐containing composite materials (GCMs). GFMs refer to graphene and its derivatives, containing 0D graphene quantum dots (GQDs), 2D graphene (GR), graphene oxide (GO), and reduced graphene oxide (rGO), 3D graphite, etc. Among them, GQDs, GR, GO, and rGO could be obtained by chemical approaches using graphite as the starting material. GCMs are composite materials consisting of GFMs and other materials such as hydroxyapatite,^[^ ^18^ ^]^ chitosan,^[^ ^19^ ^]^ polycaprolactone,^[^ ^20^ ^]^ etc. Although GFMs and GCMs could promote cell proliferation, osteogenic differentiation, and in vivo bone tissue regeneration as reported in many studies,^[^ ^21^, ^22^, ^23^ ^]^ there are also several controversial or unclear aspects remaining, such as functional mechanisms,^[^ ^24^, ^25^ ^]^ cytotoxicity,^[^ ^26^, ^27^ ^]^ and biodegradation.^[^ ^28^, ^29^ ^]^ Therefore, it is necessary to systematically review the existing literature of bGBMs research to summarize its integral development and try to discuss the important controversial or unclear topics, which is of vital importance for future research.

CiteSpace is a powerful scientific knowledge mapping software developed by Chaomei Chen.^[^ ^30^ ^]^ It has been used in much scientometric research due to its advantages of providing quantitative analysis for academic literature and complete visualization and analysis process. CiteSpace processes literature information such as titles, abstracts, keywords, authors, countries, journals, and references to generate valuable visualized information about research status, hotspots, and trends of a scientific field, to contribute systematically and objectively insight into the research status of this field.^[^ ^31^, ^32^, ^33^, ^34^, ^35^ ^]^

This study first conducts a scientometric analysis of published literature on bGBMs indexed in the Web of Science Core Collection (WOSCC) database from 2011 to 2020. After obtaining various network atlas, the information related to yearly publication, geography, subjects, keywords, and references of bGBM is presented. According to the results of the cluster network, the concerned subjects of bGBMs are discussed in detail, such as fabrication techniques, cytotoxicity, biodegradability, osteoinductivity, and their underlying mechanism. In addition, photothermal therapy, which is considered an emerging trend in bGBMs, is also explored. Finally, the future development of this field has prospected and feasible solutions to the challenges are proposed.

## 2 Results

### 2.1 Yearly Publication

**Figure 1:**
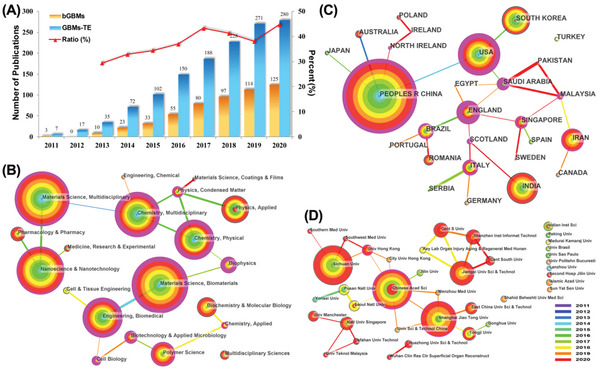
A) Number of yearly publications on bGBMs (2011–2020). B) The network of subject distribution. C) The network of contribution and cooperation of countries. D) The network of contribution and cooperation of institutions.

According to the used retrieval strategy and screening process, a dataset of a total of 540 documents is finally obtained. This dataset includes 478 original studies and 62 reviews, with a total of 22 658 cited references. We only adopt original articles and reviews because they represent the technical advances and high‐level summaries respectively and meet the requirements of CiteSpace software analysis. Other types of articles such as Meeting abstract, Book, and Correction lack some information such as keywords and references for analysis by CiteSpace, thus list in the supporting information. **Figure** **1**A shows the yearly publication of bGBMs literature. The publication of literature on graphene‐based materials (GBMs) retrieved by TS = (“tissue engineering” OR “tissue regeneration” OR “tissue repair” OR “regenerative medicine”) and TS = (“graphene”) (GBMs‐TE) are also provided. The first three published papers of bGBMs appeared in 2011.^[^ ^36^, ^37^, ^38^ ^]^ The bGBMs research entered a rapidly developing stage from 2013, since when the publication number continuously increased with time. 2019 was a milestone year for bGBMs study, with the publication number rising over 100. When compared with the publication number of GBMs‐TE, the ratio of bGBMs to GBMs‐TE was constantly over 30% from 2014, indicating that the bone‐related application was an important branch when GBMs were studied and applied for regenerative medicine.

### 2.2 Subject Categories

The subject categories of the dataset are analyzed according to the subject classification of the WOSCC database (Figure 1B). The leading subjects include “Materials Science, Multidisciplinary,” “Materials Science, Biomaterials,” “Nanoscience and Nanotechnology,” and so on. The subject “Materials Science, Coatings and Films” rise in 2020, implying a research frontier.

### 2.3 Geographical Distribution

#### 2.3.1 Contribution and Cooperation of Countries

In the cooperation network of countries (Figure 1C), a total of 27 countries publish bGBMs related literature (due to the cooperation of countries, the same article may be counted in different countries separately). The top five contributors are China, the USA, India, South Korea, and Iran. China is the significantly leading contributor in bGBMs study, accounting for 49% of the global publication. The contributions of the USA, India, and South Korea are comparable. It is noteworthy that the publication of Iran has been increasing rapidly since 2017.

The extent of cooperation between nodes is evaluated by accumulating yearly cooperation frequency. The CiteSpace automatically filters the nodes whose annual cooperation frequency is over two. The thickness of lines between nodes represents the extent of cooperation. The color of the lines marks the year when the first cooperation occurred. The betweenness centrality is displayed as the outermost purple ring of a node. A thick purple ring, i.e., high betweenness centrality, indicates that the node plays a significant connecting role. In Figure 1C, the nodes of England, Saudi Arabia, and the USA have the highest betweenness centrality.

#### 2.3.2 Contribution and Cooperation of Institutions

Regarding the publication number of institutions (Figure 1D), Sichuan University, Chinese Academy of Sciences, and Shanghai Jiao Tong University from China are the most prolific institutions in bGBMs research. Due to the great influence of geography, most of the cooperation between institutions occurs within the same country. Chinese Academy of Sciences has the most extensive cooperation with other institutions and the highest value of betweenness centrality, revealing its dominant position in bGBMs study.

### 2.4 Keywords Analysis

#### 2.4.1 Frequency and Burst of Keywords

**Figure 2:**
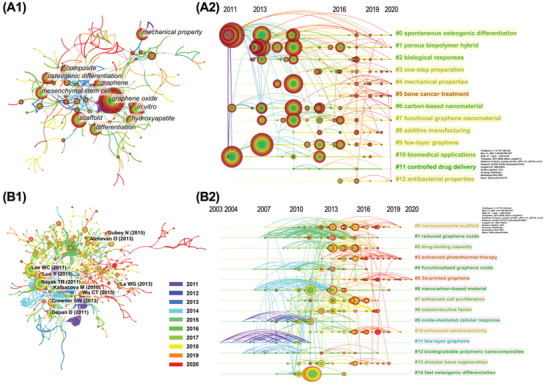
A) The network of co‐occurrence keywords. B) The network of cocitation references.

The node networks of keywords are shown in **Figure** **2**A1 and A2. The thickness of the color ring in each node is proportional to the total frequency of a keyword in the dataset. The thickness of lines between nodes evaluates the extent of co‐occurrence. Co‐occurrence refers to several keywords that appear in the same publication. The consensus is that the high extent of co‐occurrence indicates the close relationship between keywords. The central red ring of a node denotes a burst of frequency over a certain period, which could reflect the time‐changing research hotspots. The burst strength calculated by the software is proportional to the thickness of the red ring.

**Figure 3:**
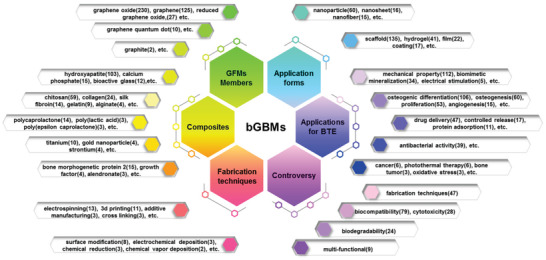
Classification of the 235 keywords according to the themes of GFMs members, composites, fabrication techniques, application forms, applications for BTE, and controversy. The frequency of keyword occurrences is numerically marked in parentheses.

In Figure 2A1 and A2, there are a total of 235 nodes. The top ten keywords are marked within Figure 2A1. This work further classifies the 235 keywords according to themes such as GFMs members, composites, fabrication techniques, application forms, applications for BTE, and controversy (**Figure** **3**). Among the different GFMs, GO is the most important member with the highest frequency. This is probably because GO is rich in oxygen‐containing functional groups on its surface and easily dispersed in water and many other solvents. Given the possible toxicity of GFMs that are directly applied in bone regeneration, GFMs are often combined with other substrates, such as ceramics, polymers, and metals, to form more feasible GCMs. Since bone is a composite mainly composed of hydroxyapatite and collagen, “hydroxyapatite” is the most emphasized biomaterial. As for the fabrication techniques of bGBMs, there are many methods mentioned in the dataset. The preparation methods of GCMs include “electrostatic spinning,” “3D printing” and so on. The application forms are mainly “scaffold,” “hydrogel,” and “film.” Considering the dispersion in physiological solutions, GFMs are mostly surface modified in the form of “nanoparticles” and “nanosheets.” In the application of BTE, the main advantages of GFMs are “mechanical property” and “osteogenic differentiation.” In addition, bGBMs are also used for drug delivery and antibacterial. In 2019–2020, there are many keywords related to bone tumor therapy, such as “photothermal therapy.” In addition, there are some keywords with high frequency but opposed to one another, such as “biocompatibility” and “cytotoxicity.” Even the same keyword like “biodegradation” was reported having conflicting research results in different literature. These keywords that are important but have no clear conclusions or criteria are included in the controversy hexagon, which will be dealt with in detail in the discussion section.

Table S1 (Supporting Information) further shows 11 keywords with strong detected bursts. The keywords “proliferation” and “carbon nanotube” are the earliest research hotspot, which burst from 2011 to 2015. Then keywords like “cellular response,” “nano hydroxyapatite,” and “expression” burst during 2013–2016, representing the intermediate hotspots. The keywords “biomineralization” and “polycaprolactone” burst at the late stage, showing the frontier attention in bGBMs study.

#### 2.4.2 Co‐Occurrence Cluster Analysis

The nodes in Figure 2A2 are clustered according to the co‐occurrence relationship. There are altogether 13 clusters screened by the software and displayed by color blocks. The detailed information of the clusters is listed in **Table** **1**. The clustering extent of the network is evaluated by a modularity value (from 0 to 1, the larger the modularity value, the greater the difference between clusters). For CiteSpace, the clustering structure is regarded as significant when the modularity value is over 0.3. The silhouette value ranging from −1 to 1 measures the similarity of the content of references in a cluster (the higher the silhouette value, the higher the similarity). The clustering result is considered reasonable and highly reliable when the modularity value and silhouette value are over 0.5 and 0.7, respectively. The modularity value and silhouette value of Figure 2A2 are found to be 0.7361 and 0.9175, meaning that the present clustering is acceptable. The largest three clusters are “spontaneous osteogenic differentiation,” “porous biopolymer hybrid,” and “biological responses,” which represent the mainstream research of bGBMs. Cluster #5 “bone cancer treatment” reveals the research frontier of bGBMs. In addition, clustering #3 “one‐step preparation,” #4 “mechanical properties,” and #8 “additive manufacturing” are also cutting‐edge researches in this field.

**Table 1:** The 13 clusters of co‐occurrence keywords

Cluster ID	Size	Silhouette	Mean [year]	Label (LLR)
0	25	0.869	2016	Spontaneous osteogenic differentiation
1	24	0.951	2014	Porous biopolymer hybrid
2	21	0.979	2014	Biological responses
3	19	0.921	2018	One‐step preparation
4	19	0.852	2016	Mechanical properties
5	18	0.865	2016	Bone cancer treatment
6	17	0.894	2017	Carbon‐based nanomaterial
7	16	0.871	2016	Functional graphene nanomaterial
8	16	0.927	2017	Additive manufacturing
9	15	0.898	2014	Few‐layer graphene
10	15	0.976	2016	Biomedical applications
11	11	0.98	2014	Controlled drug delivery
12	9	0.842	2016	Antibacterial properties

### 2.5 Citation Analysis

#### 2.5.1 Frequency and Burst of Citation

In this study, the dataset has a total number of 22 658 cited references. The network of references consists of 483 nodes and 1072 connections (Figure 2B1 and B2), where each node represents a reference and the size is proportionate to the cited frequency. The top ten cited references are marked with black text labels (Figure 2B1), and the detailed information is listed in Table S2 (Supporting Information). These references mainly focus on the effects of GFMs or GCMs on growth and osteogenic differentiation of stem cells, as well as protein delivery, providing an inspiring knowledge base for the bGBMs research.

The node with a central red ring denotes a citation burst, which means that a reference is cited more frequently than other comparable references in the same period. The citation burst is indirectly reflecting the research trend in bGBMs during a certain period. A total of 66 references with citation bursts are detected by the CiteSpace software, among which the citation bursts during 2018–2020 are listed (Table S3, Supporting Information), mainly report the promotion of mechanical properties and bioactivity of BTE scaffold by GCMs.

#### 2.5.2 Co‐Occurrence Cluster Analysis

Cocitation means that two or more references are cocited by one or more literature from the dataset. For CiteSpace, the more times the references are cocited, the more similar the research content of the references are. The color of lines between nodes indicates the year of the first cocitation. The nodes in Figure 2B2 are clustered according to the cocitation relationship. There are altogether 15 clusters screened by the software and displayed by color blocks. The detailed information of the clusters is listed in **Table** **2**. The label term of each cluster is extracted from titles of the citing literature in the dataset.^[^ ^39^ ^]^ The label color means the mean year of the cited references in the cluster. The modularity value and silhouette value of Figure 2B2 are found to be 0.7967 and 0.9044, meaning the present clustering is acceptable. The largest three clusters are “nanocomposite scaffold,” “reduced graphene oxide,” and “drug‐eluting capacity,” which represent mainstream researches of bGBMs. Cluster #3 “enhanced photothermal therapy” and #5 “3D‐printed graphene” reveal the research frontiers of bGBMs.

**Table 2:** The 15 clusters of cocitation references

Cluster ID	Size	Silhouette	Mean [year]	Label (LLR)
0	45	0.845	2014	Nanocomposite scaffold
1	42	0.957	2011	Reduced graphene oxide
2	40	0.922	2013	Drug‐eluting capacity
3	38	0.855	2016	Enhanced photothermal therapy
4	35	0.928	2011	Functionalized graphene oxide
5	35	0.895	2017	3D‐printed graphene
6	34	0.772	2011	Nanocarbon‐based material
7	33	0.923	2014	Enhanced cell proliferation
8	32	0.924	2013	Osteoinductive factor
9	32	0.932	2009	Oxide‐mediated cellular response
10	29	0.91	2015	Enhanced osteoinductivity
11	27	0.912	2008	Few‐layer graphene
12	24	0.943	2010	Biodegradable polymeric nanocomposites
13	23	0.857	2013	Alveolar bone regeneration
14	14	0.99	2010	Fast osteogenic differentiation

Timelines between nodes in Figure 2B2 clearly show the changes of cocitation over time, and, to some extent, also reflect the development track of bGBMs research. The early purple and deep blue lines are concentrated in clusters of #11, #9, and #12. The light blue and green lines are mainly distributed in clusters of #4, #6, #1, and #2. Recent yellow and red lines focus on clusters of #3, #5, #10, and #13. Fabrication of bGBMs has evolved from “few‐layer, biodegradable” to “reduced, functionalized,” and further to “3D printing.” The study of the biological performance of bGBMs has developed from “oxide‐mediated cellular response” to “drug‐eluting capacity,” and further to “photothermal therapy” and “osteoinductivity.”

## 3 Discussion

Graphene‐based materials (GBMs) play an increasingly important role in bone regeneration due to their unique properties. In this paper, we review the development of bGBMs study from 2011 to 2020 via CiteSpace software. Information such as yearly publication, subject distribution, country, and institutes contribution, cooperation network, keywords network, and reference network is systematically presented and analyzed. Despite the advantages such as excellent mechanical properties, osteogenesis, angiogenesis, drug delivery, antibacterial, etc. of bGBMs by a great number of literature included in the dataset of CiteSpace, we find that there still exists a lot of outstanding issues about bGBMs, such as standards in fabrication techniques and quality evaluation, cytotoxicity and cytocompatibility, long‐term stability of implants, and biodegradability. These are important challenges that need to be further testified and standardized before the clinical application can be attempted. Several excellent reviews also partially deal with these challenges.^[^ ^12^, ^40^, ^41^, ^42^, ^43^, ^44^, ^45^, ^46^, ^47^ ^]^ In this work, the challenges in the preparation of GFMs and the mechanisms behind the cytotoxicity, biodegradation, and osteogenesis of GFMs are emphatically analyzed and discussed. The future feasible solutions to the challenges are proposed.

### 3.1 Fabrication Techniques

The fabrication of GFMs has developed from early few‐layer GR to functionalized GO and rGO, and further to recent 3D‐printing BTE scaffolds. The development of the manufacturing technology has improved the dispersibility and stability of GFMs in physiological solutions, thus obtaining GFMS with good biocompatibility and biodegradability for biomedical applications.

Currently, the fabrication techniques of GFMs, either growing the sheets from small molecular precursors (bottom‐up) or exfoliating the bulk graphitic materials (top‐down),^[^ ^48^, ^49^, ^50^, ^51^, ^52^, ^53^ ^]^ have not been standardized. In many cases, the cytotoxicity of GFMs is found due to the residual contamination from the fabrication process.^[^ ^54^ ^]^ For example, GR prepared by CVD might be contaminated by toxic metal ions when transferred from the metallic carrier.^[^ ^55^ ^]^ And residual manganese‐containing reagent in the preparation of GO by the most established modified Hummer's method is highly toxic to cells.^[^ ^54^ ^]^ So the purity of synthetic GFMs must be carefully controlled during preparation. After preparation, a good dispersion and chemical stability of GFMs are very important for further use.^[^ ^56^, ^57^, ^58^, ^59^ ^]^ GFMs with high reactivity will undergo biotransformation in biological fluids and change their physical and chemical properties. For instance, Liu et al.^[^ ^60^ ^]^ reported that lung fluid would transform epoxy and carbonyl groups on GO into phenolic groups, leading to aggregation and precipitation of GO sheets. Instead of the complete metabolism of well‐dispersed GFMs, the aggregated GFMs could not or only partially be metabolized.^[^ ^61^, ^62^ ^]^ Therefore, the stability and dispersion of GFMs are essential for biomedical applications, especially when exposed to biological fluids.^[^ ^63^ ^]^ It is relatively easy to obtain stable and well dispersed GO liquid solution via the most established ultrasonication.^[^ ^27^, ^64^ ^]^ In contrast, pure graphene (nonoxidized) is usually spontaneously aggregated due to Van der Waals forces and high surface free energy, which thus required chemical treatments to achieve stable sheets suspension.^[^ ^59^, ^63^ ^]^ Superficial covalent or noncovalent decoration is often used to achieve well stability and dispersion of GFMs.^[^ ^65^, ^66^, ^67^, ^68^, ^69^, ^70^ ^]^ Moreover, combining GFMs with a biomaterial substrate (to form GCMs) can significantly promote the stability of GFMs. The fabrication techniques of GCMs mainly include casting plus freeze‐drying,^[^ ^71^, ^72^, ^73^, ^74^ ^]^ electrospinning,^[^ ^75^, ^76^, ^77^, ^78^, ^79^, ^80^ ^]^ 3D printing,^[^ ^81^, ^82^, ^83^, ^84^, ^85^, ^86^, ^87^ ^]^ and coating,^[^ ^88^, ^89^, ^90^, ^91^ ^]^ etc., where GFMs are ultimately encapsulated inside or superficially fixed. Compared with the coating situation, GFMs in other techniques have much less direct contact with cells and usually present better cytocompatibility.^[^ ^92^ ^]^

### 3.2 Biocompatibility

In addition to the stable dispersion in physiological solutions, other factors are affecting the biocompatibility and biodegradability of GFMs as well, which must be considered for biomedical applications.

**Figure 4:**
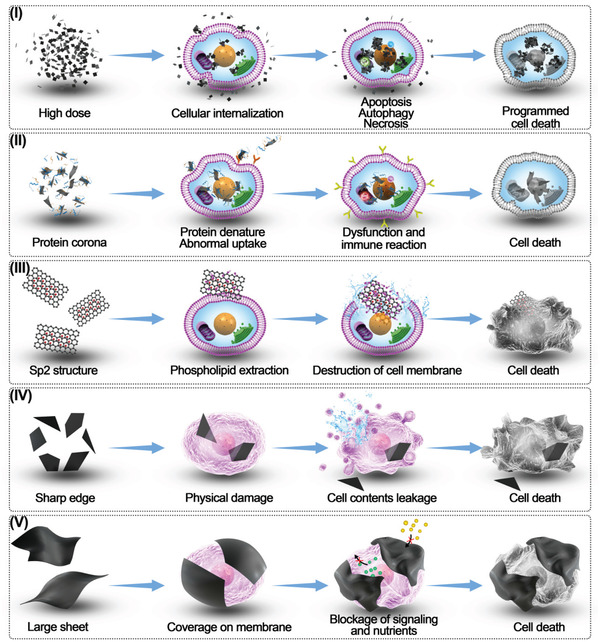
The schematic diagram of the reported cytotoxic mechanism of GFMs. I) Intracellular dysfunction. II) Protein corona. III) Extracting phospholipid. IV) Physical damage to the membrane. V) Membrane coverage.

The potential toxicity of GFMs is reported to be related to factors like size,^[^ ^93^ ^]^ dose,^[^ ^94^, ^95^ ^]^ and surface feature,^[^ ^96^ ^]^ etc. The potential toxic mechanism of GFMs include (**Figure** **4**):

I. Intracellular dysfunction. Small GFMs (less than 750 nm) will be endocytosed and then expose to the endomembrane system. The aggregated GFMs may cause nonselective physical damage to the membranes of organelles (e.g., mitochondria and endoplasmic reticulum).^[^ ^97^ ^]^ More importantly, endocytosis of GFMs will activate various signaling pathways that induce programmed cell death like apoptosis,^[^ ^98^, ^99^, ^100^ ^]^ autography,^[^ ^101^, ^102^ ^]^ and programmed necrosis.^[^ ^103^, ^104^ ^]^ For instance, GBMs induce apoptosis via organelle dysfunction, ROS generation, ER stress, and DNA damage, which will together affect MAPK and TGFB/TGF‐β signaling pathways and activate caspases.^[^ ^97^, ^98^, ^99^, ^105^, ^106^ ^]^ GBMs are also able to cause accumulation of autophagosomes and subsequent autophagic cell death via PI3K/Akt/MTOR pathway.^[^ ^101^, ^102^, ^105^, ^107^ ^]^ GBMs are reported to stimulate DNA damage, organelle dysfunctions as well as ROS production, which further induces programmed necrosis of cells via activation of RIPK1/RIPK3, MLKL/TNF, FASLG/FasL, and TNFSF10/TRAIL.^[^ ^103^, ^104^, ^108^ ^]^
II. Protein corona. Once GFMs contact the biological fluids, the proteins will adsorb onto the surface and form a protein corona (PC).^[^ ^109^ ^]^ The phenomenon will induce unfolding and denaturing of proteins, resulting in new epitope exposure or functional deterioration.^[^ ^110^, ^111^, ^112^, ^113^, ^114^ ^]^ GFMs‐PC complex adversely influences the interaction between GFMs and cells, such as uptake process and uptake dose.^[^ ^115^ ^]^ Intracellular GFMs‐PC complex will hinder the functions of organelles such as mitochondria and lysosome. The GFMs‐PC complex is also reported to directly stimulate abnormal proliferation and immune reaction.^[^ ^116^ ^]^
III. Extracting phospholipid. GFMs vigorously extract phospholipids from the cell membranes and cause breakage of cell membranes and reduction of cell viability.^[^ ^117^, ^118^, ^119^ ^]^ The strong attraction between GFMs and membrane lipids is largely derived from GFMs with unique sp^2^ structures.
IV. Physical damage to the membrane. The sharp edge of some GFMs will penetrate or insert into the cell membrane, resulting in structural damage and leakage of cell contents.^[^ ^120^ ^]^ This mechanism is reported in the antibacterial application of GFMs.^[^ ^121^, ^122^ ^]^
V. Membrane coverage. Larger GFMs (≈1 mm) will cover the cell membrane, causing cell death by blocking signal pathways and interfering with the uptake of nutrients.^[^ ^123^ ^]^ So far, there are still plenty of not fully understood details on the toxicity of GFMs, such as uptake, distribution, metabolism, clearance, and related signaling pathways.

It should also be noted that the toxicity of GFMs may be derived from the combination of the above mechanisms. Moreover, the cytotoxic mechanism of different GFMs may be different. For instance, hydrophilic GO is reported to be more likely to cause intracellular dysfunction.^[^ ^120^ ^]^ Although derived from GO, GQDs hardly triggered ROS signals but will disturb the redox‐sensitive system via selectively inhibiting the activity of the endogenous antioxidant enzyme.^[^ ^124^ ^]^ Hydrophobic rGO tends to aggregate onto the cell surface and cause physical damage to or adversely cover the cell membrane.^[^ ^125^ ^]^ Different cell types may also present different sensitivity to GFMs. For example, tumor cells have an “enhanced permeability and retention” (EPR) effect, which facilitates the accumulation of GFMs in tumor cells.^[^ ^126^ ^]^ In addition, tumor cells intrinsically possess higher levels of ROS than normal cells, which makes them more susceptible to ROS‐induced cell death.^[^ ^104^ ^]^ Therefore, a dose of GFMs that is safe for normal cells may be toxic to tumor cells.^[^ ^127^ ^]^ Immune cells like macrophages are more sensitive to GFMs than other cells like epithelial cells and stem cells.^[^ ^95^, ^128^, ^129^ ^]^ Namely, GFMs have a more obvious toxic effect on macrophages.^[^ ^130^ ^]^

Based on the above potential toxic mechanism of GFMs, researchers have exploited various methods to reduce the cytotoxicity of GFMs in the studies of the dataset.

I. Size control: Plenty of studies emphasized that the size of nanomaterials is a key feature in determining the extent of cytotoxicity.^[^ ^131^ ^]^ For example, GQDs generally have a size (2–10 nm) similar to that of proteins.^[^ ^132^ ^]^ The in vivo studies reported that GQDs could be rapidly cleared through the mouse kidney, while GO (10–30 µm) would accumulate in vivo and even lead to the death of the mouse.^[^ ^133^ ^]^ Beyond the basic dataset, larger GO (1–5 µm) showed more obvious cytotoxicity than small GO (400–700 nm).^[^ ^134^ ^]^ In contrast, the small GR and GO (30 nm) seemed more toxic than the larger counterparts (300 nm).^[^ ^26^ ^]^ There is possibly a size window within which GFMs presented minimum cytotoxicity.^[^ ^135^ ^]^
II. Dose control: A number of research revealed the dose‐dependent effects of GFMs on the survival rate of cells.^[^ ^26^, ^136^, ^137^, ^138^, ^139^ ^]^ Even the easily biodegradable GQDs would result in nuclear damage and DNA cleavage if accumulated inside the nucleus at a high dose.^[^ ^140^ ^]^ Researchers tended to reduce the dose of GFMs as much as possible only if the advantages of GFMs could be retained.^[^ ^95^, ^141^ ^]^
III. Surface modification: Surface modification is a common strategy to achieve the cytocompatibility of GFMs. Covalent functionalization of GFMs could be achieved by chemical attachment of sulfonated polyether ether ketone (SPEEK), polyethylene glycol (PEG), chitosan (CS), etc.^[^ ^19^, ^67^, ^79^, ^96^, ^142^, ^143^, ^144^ ^]^ Noncovalent functionalization is mainly conducted by adsorption of biomolecules such as proteins, peptides, and vitamins through van der Waals force, electrostatic force, and hydrogen force, etc.^[^ ^68^, ^145^, ^146^ ^]^
IV. Contact limitation: Combining GFMs with other biomaterials significantly reduces direct contact between GFMs and cells. The final composite forms include 3D porous scaffold,^[^ ^82^, ^146^ ^]^ film,^[^ ^147^, ^148^ ^]^ hydrogel mass,^[^ ^149^, ^150^ ^]^ and so on.
V. Reducing oxidation levels: The surface oxidation state is reported to be related to the toxicity of GO for mammalian cells.^[^ ^120^ ^]^ Low oxidation of graphene nanoparticles (≈88% carbon and 4.5% oxygen) could produce less ROS and maintain the biological activity of stem cells.^[^ ^151^ ^]^

### 3.3 Biodegradability

In addition to cytocompatibility, biodegradability is another important feature for the application of bGBMs. Among the basic dataset, several studies dealt with the degradation of bGBMs. Yang et al.^[^ ^152^ ^]^ reported that the addition of GO accelerated the degradation of the poly‐l‐lactic acid (PLLA) scaffold and the degradation rate was proportional to the amount of GO. This might be because GO promoted the penetration of water molecules into the PLLA matrix. Pazarceviren et al.^[^ ^153^ ^]^ reported that GBR membranes composed of gelatin and GO displayed a remarkably lower degradation rate than pure gelatin, proving that GO could improve the overall stability of GBR membranes. However, these publications do not explore the biodegradation process of GFMs. Although GFMs are once generally considered undegradable, in recent years, researchers (from literature beyond the basic dataset) found out that GFMs could be degraded by peroxidases produced by immune cells like macrophages and neutrophils.

**Figure 5:**
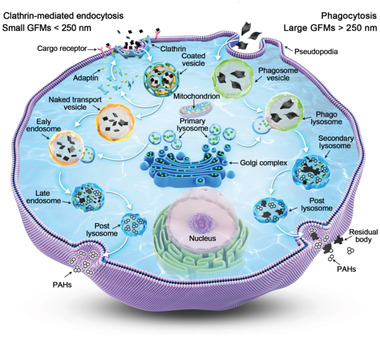
The schematic diagram of the reported biodegradation mechanism of GFMs by macrophage.

The reported in vitro biodegradation of GFMs is mainly evaluated by using macrophages (**Figure** **5**). The dispersed small GFMs (<250 nm) are easily entered into macrophages through clathrin‐mediated endocytosis.^[^ ^127^ ^]^ After being endocytosed, GFMs are transported by early endosomes and will become the target of lysosomes originating from the Golgi apparatus. Then GFMs will be subjected to a myeloperoxidase (MPO)‐mediated oxidative process.^[^ ^154^, ^155^ ^]^ This process transforms epoxy groups of GFMs to more energetically favorable carbonyl groups and results in the rupture of underlying C—C bonds.^[^ ^154^, ^156^, ^157^ ^]^ Finally, GFMs are digested into oxidized polycyclic aromatic hydrocarbons (PAHs) and carbon dioxide,^[^ ^155^, ^158^, ^159^ ^]^ and exported from macrophages via exocytosis. The dispersed large GFMs (>250 nm) will trigger invagination of membrane and be engulfed into phagosome vesicles of macrophages.^[^ ^56^ ^]^ The phagosome vesicles fused with lysosomes, where GFMs will be degraded into smaller porous sheets and oxidized PAHs by MPO.^[^ ^160^, ^161^ ^]^ These degradation products will finally be exported from cells. It should be noted that PAHs are supposed to be further processed into carbon dioxide via enzyme‐mediated catalytic biodegradation, although this process is still absent of precise detection.^[^ ^162^ ^]^ In addition to the intracellular pathway, GFMs are also reported to be extracellularly degraded by human neutrophils via degranulation mediated secretion of MPO or released of neutrophil extracellular traps (NETs) with intrinsic MPO.^[^ ^62^, ^160^, ^163^, ^164^ ^]^ This immune response of neutrophils to GFMs is short‐lived and then macrophages endocytose residual GFMs and worn‐out neutrophils.^[^ ^61^ ^]^

For in vivo studies, a significant fraction (>50%) of the well‐dispersed small GFMs (lateral dimension 10–50 nm) will be excreted intact through the urine within hours. The remaining GFMs are endocytosed by endothelial and podocyte cells and degraded as in macrophages.^[^ ^165^, ^166^ ^]^ The well‐dispersed larger GFMs (100–500 nm) first accumulate in the liver and then translocate to the spleen, following phagocytosis and degradation by marginal zone macrophages.^[^ ^154^ ^]^ The aggregated GFMs (>500 nm) accumulate in the lung for a long time.^[^ ^167^, ^168^ ^]^

### 3.4 Osteoinductivity

When the necessary conditions for biomedical applications–stable dispersion, biocompatibility, and biodegradability–have been met, the advantages of GFMs in BTE can be fully revealed. GFMs are reported to improve the physiochemical properties of bGBMs in many respects such as mechanical strength,^[^ ^169^, ^170^, ^171^, ^172^, ^173^ ^]^ Young's modulus,^[^ ^171^, ^174^, ^175^ ^]^ specific surface area,^[^ ^169^, ^176^ ^]^ surface roughness,^[^ ^171^, ^172^, ^177^, ^178^ ^]^ porosity,^[^ ^170^, ^171^, ^179^ ^]^ hydrophilicity,^[^ ^177^, ^180^, ^181^, ^182^, ^183^ ^]^ water retention,^[^ ^177^, ^181^, ^182^ ^]^ proteins adsorption,^[^ ^177^, ^180^, ^184^ ^]^ and biomineralization.^[^ ^175^, ^185^, ^186^, ^187^, ^188^, ^189^, ^190^ ^]^ In terms of biological performances, bGBMs promote adhesion,^[^ ^169^, ^179^, ^191^, ^192^ ^]^ proliferation,^[^ ^171^, ^172^, ^174^, ^175^, ^179^, ^193^ ^]^ and osteogenic differentiation of stem cells.^[^ ^142^, ^169^, ^171^, ^176^, ^179^, ^186^, ^191^, ^192^ ^]^ When implanted in vivo, bGBMs promote new bone formation^[^ ^178^, ^182^, ^186^, ^191^, ^194^, ^195^ ^]^ and neovascularization,^[^ ^196^, ^197^ ^]^ with no adverse inflammatory response.^[^ ^198^, ^199^ ^]^ Due to the existence of GFMs, bGBMs also present antibacterial properties.^[^ ^177^, ^200^, ^201^, ^202^, ^203^, ^204^, ^205^, ^206^ ^]^ Moreover, the introduction of GFMs realizes the sustained release of loaded drugs and bioactive molecules.^[^ ^173^, ^182^, ^184^, ^207^, ^208^, ^209^, ^210^ ^]^

**Figure 6:**
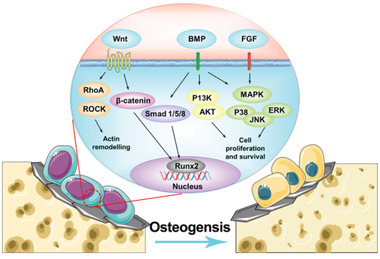
A schematic view of the complex network of the GFMs mediated signaling pathways in regulating bone regeneration.

Since many articles demonstrated that the osteogenic potential of GBMs persisted even in the absence of chemical inductors, researchers tried to explore the underlying mechanotransduction.^[^ ^43^ ^]^ Although several hypotheses have been proposed, many detailed aspects are awaiting to be clarified. The reported mechanisms up to now are as follows:

I. Binding ability with biomolecules. GFMs have a strong capability to absorb various osteogenic induction biomolecules such as dexamethasone and BMPs by hydrogen bonding or electrostatic interactions, acting as a pre‐enrichment platform of OI factors to promote differentiation of stem cells into osteoblasts.^[^ ^37^, ^211^, ^212^, ^213^ ^]^
II. Promotion of hydroxyapatite (HAp) mineralization. Negatively charged GFMs absorb Ca^2+^ and support the nucleation and growth of HAp,^[^ ^141^, ^214^, ^215^ ^]^ constructing a favorable base for osteogenesis.
III. Activation of the osteogenic signaling pathways. GFMs activate different osteogenic signaling pathways.^[^ ^43^, ^216^ ^]^ The osteoinductivity of GO occurs through the activation of Wnt/β catenin,^[^ ^93^, ^94^, ^217^, ^218^, ^219^ ^]^ ATF4 and ERK1/2,^[^ ^143^ ^]^ and JNK and FoxO1 signaling pathways of stem cells.^[^ ^24^ ^]^ GR activates the PI3K/Akt/GSK‐3β/β‐catenin,^[^ ^220^ ^]^ mechanosensitive integrin/FAK axis,^[^ ^25^, ^217^, ^221^, ^222^ ^]^ and ERK/MAPK^[^ ^223^, ^224^, ^225^, ^226^, ^227^ ^]^ signaling pathways to promote osteogenesis of stem cells.
IV. Activation of the angiogenic signaling pathway. It was reported that GO in bioglass scaffolds could promote the expression of VEGF and HIF‐1α of MSCs by activating Akt/eNOS signaling pathway.^[^ ^216^, ^228^ ^]^ GO also stimulate VEGF expression of RAW264.7 cells via the TLR/NF‐κB signaling pathway and the corresponding conditioned medium enhances the angiogenesis of HUVECs.^[^ ^224^ ^]^ The introduction of GR into calcium silicate scaffold promotes osteogenesis and vascularization of MSCs through FGFR pathway.^[^ ^229^ ^]^ A schematic view of the complex network of the signaling pathways regulating bone regeneration is proposed in **Figure** **6**.

### 3.5 Photothermal Therapy

**Figure 7:**
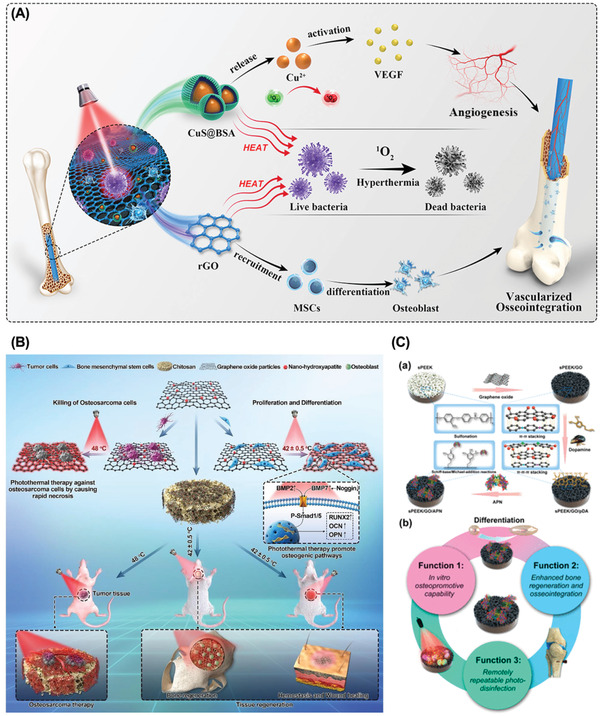
A) Schematic illustration of synergistic photocatalytic antibacterial and osseointegration via coupling CuS@BSA NPs and rGO. Reproduced with permission.^[^ ^232^ ^]^ Copyright 2021, Elsevier Ltd. B) Schematic illustration of the fabrication of nHA/GO particles, nHA/GO/CS scaffolds, and their bioapplication. Reproduced with permission.^[^ ^230^ ^]^ Copyright 2020, Elsevier Ltd. C) Schematic illustration of the fabrication of the multifunctional sPEEK a) and its triple‐model therapeutic effects b). Reproduced with permission.^[^ ^202^ ^]^ Copyright 2020, ACS Publications.

Besides osteogenic and mechanical properties, the inclusion of GFMs also enhances the optical properties and the light‐to‐heat conversion efficiency of materials, which endowed bone‐regenerating biomaterials with therapeutic functionality, such as bone–tumor therapy/regeneration^[^ ^230^, ^231^ ^]^ and bone antibacterial therapy/regeneration.^[^ ^202^, ^232^ ^]^ (**Figure** **7**)

Phototherapy such as photodynamic therapy (PDT) and photothermal therapy (PTT) in cancer treatment has developed quickly over the past few years. In photodynamic therapy, irradiation of a specific wavelength will activate photosensitizing drugs concentrated in the tumor tissue. This will transfer energy to the surrounding oxygen, producing highly active singlet oxygen (^1^O_2_). Singlet oxygen can oxidize nearby biomacromolecules, producing cytotoxicity and killing tumor cells. Therefore, adequate oxygen supply is the prerequisite to ensure the efficiency of PDT.^[^ ^233^, ^234^, ^235^ ^]^ As bone tumor has a hypoxia microenvironment, reversing tumor hypoxia is a necessary but difficult strategy in bone tumor treatment. This may be why the number of studies of bGBMs as photosensitizing drugs is relatively small in this dataset.^[^ ^236^ ^]^

PTT uses the thermal energy induced by light‐to‐heat conversion materials to kill cancer cells.^[^ ^237^ ^]^ The core principle of PTT is to convert light absorbed by highly efficient photothermal agents (PTAs) in the target tissue into heat energy through external near‐infrared (NIR) light, thereby increasing the local temperature to 45–50 °C for a short period. Heat induces irreversible damage on cells, causing protein denaturation, the collapse of cells’ membrane, and dysfunctions in the activity of enzymes and mitochondria. These events ultimately led to cell death by coagulative necrosis.^[^ ^238^, ^239^, ^240^ ^]^ In addition, the low‐power NIR facilitates wound healing, bone repair, angiogenesis, etc.^[^ ^241^, ^242^, ^243^ ^]^ In this process, the PTAs with high photothermal conversion efficiency exert the largest agglomerative thermal effect, to achieve a fixed‐point temperature rise and then heat the tumor, while the normal tissue is not damaged by heat. Therefore, the photothermal conversion efficiency of PTAs is very important.^[^ ^244^ ^]^

Compared to other nanomaterials, GFMs have a large surface‐to‐volume ratio and unique electronic structure, which transform light energy into heat energy through the photothermal effect of plasma resonance. They have been used as common PTAs in the study of PTT.^[^ ^245^ ^]^ Unlike other PTAs that may undergo structural changes after long‐term exposure to NIR, the structure of GFMs is relatively stable. In addition, the planar structure and π‐conjugated structure of GFMs allow them to immobilize a great number of substances, including drugs, genes, fluorescent probes, biomolecules, and metals.^[^ ^234^, ^244^ ^]^ Upon irradiation with a near‐infrared ray, the photon energy is converted into heat via nonradiative decay transitions, which can be used to kill tumor cells for PTT. Meanwhile, the noncovalent bond interactions on the surface of GFMs diminished due to the absorption of light, increased temperature and atomic vibrations. Thus, the drugs could be rapidly released from the surface of GFMs. Among the dataset, after exposure to NIR light for some time, bGBMs have been reported to have therapeutic properties of inhibiting tumor cells,^[^ ^230^, ^231^, ^246^, ^247^ ^]^ drug delivery,^[^ ^248^ ^]^ and antimicrobial activity.^[^ ^202^, ^232^, ^249^, ^250^ ^]^ (**Table** **3**)

**Table 3:** PTT applications of bGBMs

bGBMs	Laser parameters	Targeted temperature	Therapeutic effect under NIR	Bone regeneration		Ref.
				Under NIR	Without NIR	
CuS/rGO	808 nm, 2 W cm^−2^, 600 s	52.3 °C	Antimicrobial	–	Promoted vascularized osseointegration	^[^ ^232^ ^]^
GR/HA/Gelatin	808 nm, 1 W cm^−2^, 180 s	43 °C.	–	Mild photothermal environment accelerated bone regeneration.	–	^[^ ^242^ ^]^
CS/rGO loading Teriparatide	808 nm, 0.5 W cm^−2^, 10 min	48 °C	Trigger delivery of anticancer drug	–	Improved osteoporotic bone defect repair	^[^ ^248^ ^]^
CePO4/CS/GO	In vitro: 808 nm, 4.6 W cm^−2^, 5 min; In vivo: 0.55 W cm^−2^, 10 min	In vitro: 51.4 °C; In vivo: 52 °C	Anticancer	–	Regulated macrophage polarization to improve the osteoinductive ability	^[^ ^246^ ^]^
GO/pDA	808 nm, 0.5 W/cm^−2^, 10 min	47.6 °C	Antimicrobial	–	Boosted in vivo osseointegration and bone remodeling	^[^ ^249^ ^]^
nHA/GO/CS	808 nm, 2 W cm^−2^, 5 min; 1 W cm^−2^, 60 s	2 W cm^−2^, 48 °C, 1 W cm^−2^, 42 °C	Anticancer	Promoting osteogenesis, hemostasis, and soft tissue repair under irradiation	–	^[^ ^230^ ^]^
PEEK/GO	808 nm, 0.43 W cm^−2^, 150 s	45 °C	Anticancer and Antimicrobial	–	Promoted new bone tissue formation.	^[^ ^251^ ^]^
sPEEK/GO/APN	808 nm, 0.5 W cm^−2^, 10 min	50.4 °C	Antimicrobial	–	Boosted bone regeneration and osseointegration	^[^ ^202^ ^]^
CS/GO	808 nm, 0.66 W cm^−2^, 10 min	55 °C	Anticancer	–	Promoted the proliferation and differentiation of MC3T3‐E1	^[^ ^247^ ^]^
CS/nHA/CD	808 nm, 1 W cm^−2^, 10 min	51.4 °C	Anticancer and Antimicrobial	–	Enhanced osteoinductivity	^[^ ^252^ ^]^
nHA‐rGO	808 nm, 1.0 W cm^−2^, 10 min	77 °C	Anticancer	–	Promoted large‐format bone defect repair	^[^ ^231^ ^]^
GO‐TCP	808 nm, 0.36 W cm^−2^, 10 min	52 °C	Anticancer	–	Promoted new bone formation	^[^ ^250^ ^]^

The photothermal effects of GFMs are related to their size, concentration, and aromaticity. Compared with GO, rGO showed preferable conductive and optical absorbance,^[^ ^231^ ^]^ which might be explained as follows: rGO restores the nanostructures of aromatic lattice, thus improving their NIR absorption and photothermal potential. In addition, coupling GFMs with organic/inorganic PTAs can reinforce their photothermal performance.^[^ ^232^ ^]^

### 3.6 Outlook

Based on the above analysis and summary, we expect that bGBMs will have a good application prospect because of their excellent properties, such as large specific surface area, easily modified functional groups, excellent mechanical properties, electrical conductivity, photothermal conversion performance, promoting osteogenesis and angiogenesis, antibacterial, drug delivery, etc. However, there are still many issues need to be solved before the broad application of bGBMs.

First, there is a lack of unified standards for the fabrication techniques and quality evaluation of GFMs. It is difficult to compare the evaluation results of biological properties of GFMs due to the inconsistency in dose, shape, surface chemistry, exposure route, and purity of GFMs used in different works. The majority of existing studies are accompanied by the difficulty of decoupling the effects of different properties of GFMs. Definite information on the specific effect of the individual property is scarce. The first step to solve this problem is to guarantee that bGBMs are prepared and evaluated based on reliable standard schemes. This is also the prerequisite for future regulatory approval and clinical applications. Second, there are few studies focused on exploring the mechanisms behind the biological properties of GFMs. After implementing the standardization of GFMs fabrication and assessment, more in‐depth and systematic researches are needed to reveal the relationship between each physicochemical characteristic of GFMs and cellular response, either separately or in combination. Finally, as bone defect regeneration spans from months to years, the current GFMs studies lack of long‐term in vivo assay, including the long‐term stability of GFMs in vivo (especially in a specific physiological environment or a changing physiological environment like in PTT), and toxicity, etc.

In addition, with the development of manufacturing technology and detection technology, we believe that there will be more in‐depth and practical researches on bGBMs.

## 4 Conclusion 

CiteSpace software is used here to visually display the development rules and trends of bGBMs, and to systematically and objectively analyze the research status, hotspots, and frontiers of bGBMs. According to the results of the cluster network derived from co‐occurrence keywords and cocitation references, the fabrication of GFMs has developed from the early few‐layer GR to functionalized GO and rGO, and further to recent 3D‐printing BTE scaffolds. The development of manufacturing technology has improved the dispersibility and stability of GFMs in physiological solutions, obtaining good biocompatibility and biodegradability for possible BTE applications. The study on the biological performance of bGBMs has progressed from early oxide‐mediated cellular response to drug‐eluting capacity, and further to recent osteoinductivity and photothermal therapy. Based on the above analysis, bGBMs are moving towards being customizable and multifunctional. The remarkable biological properties of bGBMs, such as cytotoxicity, biodegradation, osteoinductivity, photothermal properties, and their underlying mechanisms are explored in detail in the discussion section. However, evaluation results of biological properties of GFMs are usually not consistent due to complex combinations of dose, shape, surface chemistry, exposure route, and purity of GFMs used in reported works. Therefore, standard guidelines in terms of the quality of GFMs are necessary for the future for both academic research and clinical application of bGBMs.

## 5 Data and Methods

### 5.1 Dataset

The Web of Science Core Collection (WOSCC) database is the most widely used source of scientific information. This study used data from the WOSCC database for two reasons. First, it provides literature that meets the format requirements of the CiteSpace software. Secondly, literature from the WOSCC database contains comprehensive citation information. We performed all the online retrieval from the WOSCC on January 26, 2021, to avoid deviations possibly caused by daily database updates. The following retrieval strategy was used: TS = (“bone tissue engineering” OR “bone regeneration” OR “bone repair” OR “osteogenesis” OR “bone formation”) and TS = (“graphene”) as the search term, “English” as the language, “2011/01/01 to 2020/12/31” as the time span (when the time span was set as “all years,” the first publication of bGBMs was found to appear in 2011), “article” and “review” as the literature category. The screening process of the retrieval strategy is shown in Figure S1 (Supporting Information). The “full record and cited references” of the screened records were extracted in the format of “plain text” for CiteSpace software.

### 5.2 Parameter Setting

The main functions of CiteSpace software (CiteSpace 5.7.R1) include yearly publication analysis, spatial distribution and cooperation analysis, subjects analysis, co‐occurrence keywords analysis, cocitation references analysis, etc. To show the evolution of the research contents more clearly, this review selected a time slice of one year. During each time slice, we selected the top 50 nodes in the cooperation network of countries, the top ten nodes in the cooperation network of institutions, and the top 20 nodes in the subject categories. The nodes *g*‐index (*k* = 15) and *g*‐index (*k* = 25) were used in the network analysis of keywords co‐occurrence and references cocitation. The networks of each time slice were merged and then pruned by a pathfinder algorithm.

## Conflict of Interest

The authors declare no conflict of interest.

## Author Contributions

X.P. assisted in conceptualization, methodology, formal analysis, data curation, writing original draft, and visualization. D.C. assisted in validation, writing original draft, and supervision. C.R. assisted in conceptualization and supervision. Y.H. assisted in supervision. C.L. assisted in conceptualization and supervision.

## Supporting information

Supporting Information
