# Supplementary material for: Development of Graphene‐Based Materials in Bone Tissue Engineaering
Source: Glob Chall. 2021 Dec 2;6(2):2100107. doi: 10.1002/gch2.202100107 (PMC8812920; doi:10.1002/gch2.202100107)
Supplement: Supplementary file 1 — Supporting Information [file GCH2-6-2100107-s001.pdf]

Global Challenges
Open Access

Supporting Information

for *Global Challenges,* DOI: 10.1002/gch2.202100107

Development of Graphene-Based Materials in Bone Tissue Engineaering

Xiaoling Pan, Delin Cheng, Changshun Ruan, Yonglong Hong, and Cheng Lin**

Supporting Information

Development of graphene-based materials in bone tissue engineering

Xiaoling Pan, Delin Cheng, Changshun Ruan, Yonglong Hong, Cheng Lin**

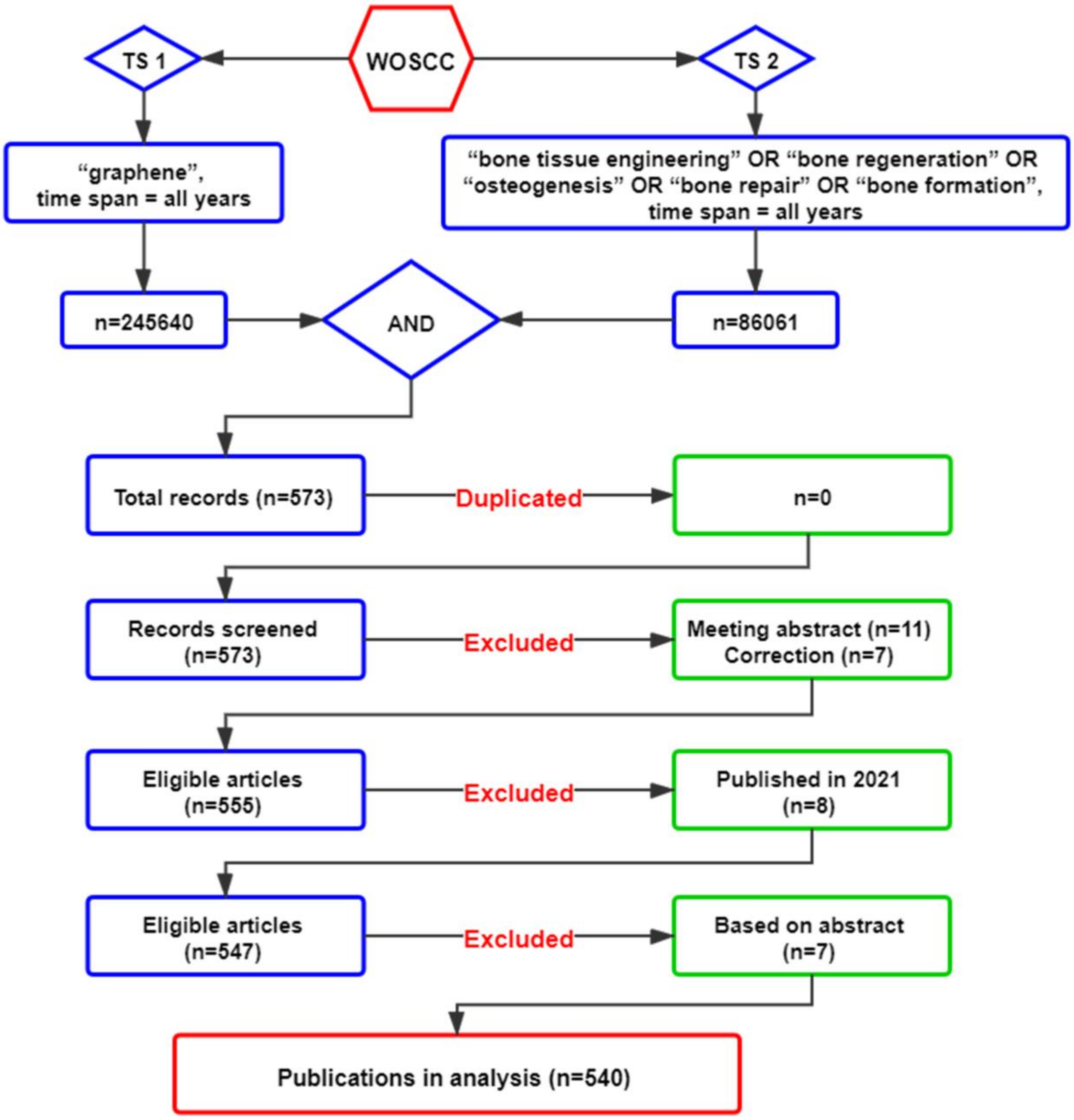

Figure S1. The retrieving and screening flowchart of literature in bGBMs. Only original articles and reviews are included, and other types of articles excluded are listed below.[1-18]

Table S1. The keywords with the strongest bursts.

Keywords	Strength	Duration<br>2011 2015 2020
proliferation	2.9669	
carbon nanotube	5.4349	
film	5.0407	
cellular response	3.3805	
growth	4.8735	
stromal cell	2.5028	
graphite oxide	3.601	
nano hydroxyapatite	2.7804	
expression	3.2501	
biomineralization	2.8667	
polycaprolactone	2.9382	

Table S2. The top ten most cited references.

Frequency	References	Tittle	Cluster #	Ref.
124	Lee WC, 2011, ACS NANO, 5, 7334	Origin of enhanced stem cell growth and differentiation on graphene and graphene oxide	14	[19]
115	Nayak TR, 2011, ACS NANO, 5, 4670	Graphene for controlled and accelerated osteogenic differentiation of human mesenchymal stem cells	14	[20]
58	Luo Y, 2015, ACS APPL MATER INTER, 7, 6331	Enhanced proliferation and osteogenic differentiation of mesenchymal stem cells on graphene oxide-incorporated electrospun poly(lactic-co-glycolic acid) nanofibrous mats	7	[21]
56	Depan D, 2011, ACTA BIOMATER, 7, 3432	Structure-process-property relationship of the polar graphene oxide-mediated cellular response and stimulated growth of osteoblasts on hybrid chitosan network structure nanocomposite scaffolds	6	[22]
47	Crowder SW, 2013, NANOSCALE, 5, 4171	Three-dimensional graphene foams promote osteogenic differentiation of human mesenchymal stem cells	6	[23]
45	Kalbacova M, 2010, CARBON, 48, 4323	Graphene substrates promote adherence of human osteoblasts and mesenchymal stromal cells	14	[24]
42	La WG, 2013, SMALL, 9, 0	Delivery of a therapeutic protein for bone regeneration from a substrate coated with graphene oxide	0	[25]
42	Alzhavan O, 2013, CARBON, 59, 200	Graphene nanogrids for selective and fast osteogenic differentiation of human mesenc hymal stem cells	2	[26]
42	Dubey N, 2015, STEM CELLS INT, 0, 0	Graphene: A versatile carbon-based material for bone tissue engineering	2	[27]
41	Wu CT, 2015, CARBON, 93, 116	Graphene-oxide-modified beta-tricalcium phosphate bioceramics stimulate in vitro and in vivo osteogenesis	0	[28]

Table S3. The references with cited burst during 2018-2020.

References	Tittle	Strength	Duration<br>2011 2015 2020	Ref.
Lee C, 2008, SCIENCE, V321, P385,	Measurement of the elastic properties and intrinsic strength of monolayer graphene	6.8189		[29]
Geim AK, 2007, NAT MATER, V6, P183,	Detection of individual gas molecules adsorbed on graphene	6.7165		[30]
Nie W, 2017, CARBON, V116, P325,	Three-dimensional porous scaffold by self-assembly of reduced graphene oxide and nano-hydroxyapatite composites for bone tissue engineering	5.6643		[31]
Gao CD, 2017, ACTA BIOMATER, V61, P1,	Carbon nanotube, graphene and boron nitride nanotube reinforced bioactive ceramics for bone repair	5.5471		[32]
Kim S, 2011, ADV MATER, V23, P2009,	Graphene-biomineral hybrid materials	5.1021		[33]
Park SY, 2011, ADV MATER, V23, P0,	Enhanced differentiation of human neural stem cells into neurons on graphene	4.9361		[34]
Gao CD, 2017, BONE RES, V5, P0,	Bone biomaterials and interactions with stem cells	4.8503		[35]
Li M, 2013, J MATER CHEM B, V1, P475,	In situ synthesis and biocompatibility of nano hydroxyapatite on pristine and chitosan functionalized graphene oxide	4.8118		[36]
Sun XM, 2008, NANO RES, V1, P203,	Nano-graphene oxide for cellular imaging and drug delivery	4.8047		[37]
Kim J, 2013, J MATER CHEM B, V1, P933,	Graphene-incorporated chitosan substrata for adhesion and differentiation of human mesenchymal stem cells	4.5143		[38]

Reference

[1] Ali, A.; Bano, S.; Priyadarshi, R.; Negi, Y. S. In *Effect of carbon based fillers on properties of Chitosan/PVA/beta TCP based composite scaffold for bone tissue engineering*, International conference on BioMaterials, BioEngineering and BioTheranostics (BioMET), Vellore, INDIA, Jul 24-28; Elsevier Science Bv: Vellore, INDIA, **2018**.

[2] Arnold, A.; Holt, B.; Sydlik, S., *Abstracts of Papers of the American Chemical Society* **2016,** *252*, 1.

[3] Caetano, G. F.; Wang, W.; Chiang, W. H.; Cooper, G.; Diver, C.; Blaker, J. J.; Frade, M. A.; Bartolo, P., *3d Printing and Additive Manufacturing* **2019,** *6* (4), 234.

[4] Choe, G.; Ieee, Three-Dimensional Mesenchymal Stem Cell Printing and Bone Regeneration Using Graphene Oxide/Alginate Composites. In *2019 13th Ieee International Conference on Nano/Molecular Medicine & Engineering*, Ieee: New York, 2019.

[5] Dhar, M.; Elkhenany, H.; Bourdo, S.; Alghazali, K.; Biris, A.; Anderson, D., *Tissue Engineering Part A* **2017,** *23*, S87.

[6] Dinescu, S.; Ignat, S. R.; Ionita, M.; Radu, E.; Jianu, D.; Costache, M., *Febs Journal* **2017,** *284*, 380.

[7] Ding, X. L.; Kang, H. Y.; Zou, T. Q.; Liu, H. F.; Fan, Y. B., *Journal of Controlled Release* **2017,** *259*, E153.

[8] Eqtesadi, S.; Motealleh, A.; Wendelbo, R.; Ortiz, A. L.; Miranda, P., *Journal of the European Ceramic Society* **2017,** *37* (12), 3695.

[9] Jabbarnia, A.; Patlolla, V. R.; Misak, H. E.; Asmatulu, R.; Asme In *ELECTROSPUN FIBERS INCORPORATED WITH HYDROXYAPATITE NANOPARTICLES AND GRAPHENE NANOFLAKES FOR BONE SCAFFOLDING*, ASME International Mechanical Engineering Congress and Exposition, Houston, TX, Nov 09-15; Amer Soc Mechanical Engineers: Houston, TX, **2012**.

[10] Khiabani, A. B.; Rahimi, S.; Yarmand, B.; Mozafari, M. In *Electrophoretic deposition of graphene oxide on plasma electrolytic oxidized-magnesium implants for bone tissue engineering applications*, INN International Conference/Workshop on Nanotechnology and Nanomedicine (NTNM), Mat & Energy Res Ctr, Tehran, IRAN, May 02-03; Elsevier Science Bv: Mat & Energy Res Ctr, Tehran, IRAN, **2017**.

[11] Kumar, S.; Azam, D.; Raj, S.; Kolanthai, E.; Vasu, K. S.; Sood, A. K.; Chatterjee, K., *Journal of Biomedical Materials Research Part B-Applied Biomaterials* **2016,** *104* (4), 732.

[12] Selaru, A.; Dinescu, S.; Becheru, D.; Ignat, S.; Lazar, A.; Samoila, I.; Radu, E.; Ionita, M.; Costache, M., *FEBS Open Bio* **2019,** *9*, 239.

[13] Shin, Y. C.; Kang, S. H.; Lee, J. H.; Kim, B.; Hong, S. W.; Han, D. W., *Journal of Biomaterials Science-Polymer Edition* **2018,** *29* (7-9), 762.

[14] Sydlik, S.; Arnold, A.; Hold, B.; Wright, Z., *Abstracts of Papers of the American Chemical Society* **2016,** *251*, 1.

[15] Techaniyom, P.; Sirivisoot, S.; Acm In *Enhanced Osteoblastic Differentiation Using Graphene Oxide Coating on Anodized Titanium*, 5th International Conference on Biomedical and Bioinformatics Engineering (ICBBE), Okinawa Inst Sci & Technol Grad Univ, Okinawa, JAPAN, Nov 12-14; Assoc Computing Machinery: Okinawa Inst Sci & Technol Grad Univ, Okinawa, JAPAN, **2018**.

[16] Wang, W. G.; Chiang, W. H.; Bartolo, P. J. In *DESIGN, FABRICATION AND EVALUATION OF PCL/GRAPHENE SCAFFOLDS FOR BONE REGENERATION*, 2nd International Conference on Progress in Additive Manufacturing (Pro-AM), Nanyang

Technolog Univ, SINGAPORE, May 16-19; Research Publishing Services: Nanyang Technolog Univ, SINGAPORE, **2016**.

[17] Zhang, W. J.; Chang, Q.; Xu, L.; Li, G. L.; Yang, G. Z.; Ding, X.; Wang, X. S.; Cui, D. X.; Jiang, X. Q., *Advanced healthcare materials* **2019,** *8* (5), 1.

[18] Zhu, B. T.; Wu, H. Y.; Tu, S. C.; Li, S. Q.; Liu, J. G.; Wei, J. C.; Ding, J. X.; Zhuang, X. L., *Journal of Controlled Release* **2017,** *259*, E12.

[19] Lee, W. C.; Lim, C.; Shi, H.; Tang, L. A. L.; Wang, Y.; Lim, C. T.; Loh, K. P., *ACS nano* **2011,** *5* (9), 7334.

[20] Nayak, T. R.; Andersen, H.; Makam, V. S.; Khaw, C.; Bae, S.; Xu, X. F.; Ee, P. L. R.; Ahn, J. H.; Hong, B. H.; Pastorin, G.; Ozyilmaz, B., *ACS nano* **2011,** *5* (6), 4670.

[21] Luo, Y.; Shen, H.; Fang, Y. X.; Cao, Y. H.; Huang, J.; Zhang, M. X.; Dai, J. W.; Shi, X. Y.; Zhang, Z. J., *ACS applied materials & interfaces* **2015,** *7* (11), 6331.

[22] Depan, D.; Girase, B.; Shah, J. S.; Misra, R. D. K., *Acta biomaterialia* **2011,** *7* (9), 3432.

[23] Crowder, S. W.; Prasai, D.; Rath, R.; Balikov, D. A.; Bae, H.; Bolotin, K. I.; Sung, H. J., *Nanoscale* **2013,** *5* (10), 4171.

[24] Kalbacova, M.; Broz, A.; Kong, J.; Kalbac, M., *Carbon* **2010,** *48* (15), 4323.

[25] La, W. G.; Park, S.; Yoon, H. H.; Jeong, G. J.; Lee, T. J.; Bhang, S. H.; Han, J. Y.; Char, K.; Kim, B. S., *Small (Weinheim an der Bergstrasse, Germany)* **2013,** *9* (23).

[26] Alzhavan, O.; Ghaderi, E.; Shahsavar, M., *Carbon* **2013,** *59*, 200.

[27] Dubey, N.; Bentini, R.; Islam, I.; Cao, T.; Neto, A. H. C.; Rosa, V., *Stem cells international* **2015,** *2015*.

[28] Wu, C. T.; Xia, L. G.; Han, P. P.; Xu, M. C.; Fang, B.; Wang, J. C.; Chang, J.; Xiao, Y., *Carbon* **2015,** *93*, 116.

[29] Lee, C.; Wei, X.; Kysar, J. W.; Hone, J., *Science* **2008,** *321* (5887), 385.

[30] A. K. GEIM, K. S. N., *nature materials* **2007,** *6*, 183.

[31] Nie, W.; Peng, C.; Zhou, X. J.; Chen, L.; Wang, W. Z.; Zhang, Y. Z.; Ma, P. X.; He, C. L., *Carbon* **2017,** *116*, 325.

[32] Gao, C. D.; Feng, P.; Peng, S. P.; Shuai, C. J., *Acta biomaterialia* **2017,** *61*, 1.

[33] Kim, S.; Ku, S. H.; Lim, S. Y.; Kim, J. H.; Park, C. B., *Advanced materials (Deerfield Beach, Fla.)* **2011,** *23* (17), 2009.

[34] Park, S. Y.; Park, J.; Sim, S. H.; Sung, M. G.; Kim, K. S.; Hong, B. H.; Hong, S., *Advanced materials (Deerfield Beach, Fla.)* **2011,** *23* (36), H263.

[35] Gao, C. D.; Peng, S. P.; Feng, P.; Shuai, C. J., *Bone Research* **2017,** *5*.

[36] Li, M.; Wang, Y. B.; Liu, Q.; Li, Q. H.; Cheng, Y.; Zheng, Y. F.; Xi, T. F.; Wei, S. C., *Journal of Materials Chemistry B* **2013,** *1* (4), 475.

[37] Sun, X.; Liu, Z.; Welsher, K.; Robinson, J. T.; Goodwin, A.; Zaric, S.; Dai, H., *Nano Res* **2008,** *1* (3), 203.

[38] Kim, J.; Kim, Y. R.; Kim, Y.; Lim, K. T.; Seonwoo, H.; Park, S.; Cho, S. P.; Hong, B. H.; Choung, P. H.; Chung, T. D.; Choung, Y. H.; Chung, J. H., *Journal of Materials Chemistry B* **2013,** *1* (7), 933.
